# Young adults’ needs when seeking first-line healthcare: A grounded theory design

**DOI:** 10.1371/journal.pone.0263963

**Published:** 2022-02-15

**Authors:** Lisa Viktorsson, Eva Törnvall, Magnus Falk, Ingrid Wåhlin, Pia Yngman-Uhlin

**Affiliations:** 1 Unit for Research and Development, and Department of Health, Medicine and Caring Science, Linköping University, Linköping, Sweden; 2 Management Department in Region Östergötland, and Department of Health, Medicine and Caring Science, Linköping University, Linköping, Sweden; 3 Primary Health Care Centre Kärna, and Department of Health, Medicine and Caring Science, Linköping University, Linköping, Sweden; 4 Research Section, Region Kalmar County, Kalmar, Sweden; 5 School of Health and Caring Sciences, Linnaeus University, Växjö, Sweden; Universitá Cattolica del Sacro Cuore, ITALY

## Abstract

**Background:**

Healthcare outpatient visits have increased in recent years, and young adults are often given as an explanatory factor for many avoidable visits.

**Objective:**

The objective of this study was to explore how young adults perceive seeking first-line healthcare.

**Design and setting:**

The study utilized a grounded theory design with data collection at primary healthcare centres and emergency departments in southeast Sweden.

**Method:**

Data were collected during individual interviews and patient observations with subsequent interviews during the years 2017–2018. The analysis was performed using grounded theory.

**Results:**

The main concern when young adults are seeking healthcare is that their worries are taken seriously. It is a four-part process: becoming aware of, verifying, communicating, and receiving an opinion about one’s symptoms. The process includes external factors, clarity of symptoms, behavioural approaches, healthcare know-how, enabling self-management, and prior healthcare experience(s). When communicating symptoms, the clearer the symptoms, the less there needs to be communicated. When symptoms are unclear, the importance of different behavioural approaches and healthcare know-how increases. When receiving a medical opinion about symptoms, young adults want to learn how to self-manage their symptoms. Depending on previous healthcare experience, the healthcare visit can either harm or help the patient in their healthcare-seeking process.

**Conclusion:**

This study reflects several insights in the healthcare-seeking process from a young adult perspective. Based on the results, we suggest that healthcare providers focus on the final step in the healthcare-seeking process when giving their medical opinion about symptoms. Having extra minutes to give support for future self-care regardless of diagnosis could increase positive healthcare experiences and increase future self-care among young adults.

## Introduction

Healthcare outpatient visits have increased in recent years, and long wait times for care have become an issue in many countries [1, 2]. Therefore, potentially avoidable healthcare visits have become a topic of research. According to Parkinson et al. [3], the term ‘avoidable visit’, from the perspective of a healthcare system, can be defined on the basis of three different categories: (1) divertible, by having healthcare needs more appropriately treated elsewhere; (2) preventable, meaning that attendance could have been prevented if earlier measures were taken; and (3) unnecessary, not requiring any clinical care at all. The category can only be established in retrospect. Several studies have found that healthcare personnel consider a substantial proportion of healthcare visits to fall within the third category, i.e., to be unnecessary [4–6]. However, there is an ongoing debate regarding the appropriateness of classifying healthcare visits according to whether they are unnecessary and what factors to consider when defining them as avoidable [7]. As expected, there is a discrepancy between providers’ and patients’ opinions as to what visits should be considered unnecessary [8].

Healthcare utilisation, defined as the use of healthcare services, correlates with accessibility to these healthcare services. As described by Khan and Bhardwaj, access to healthcare is partly the availability of healthcare resources relative to the need for service and partly the actual use of resources to satisfy those needs [9]. One way of measuring access to medical care is the number of doctors per capita. Sweden has a relatively high number of doctors per capita, 4.2/1000 (2016), compared to many other European countries [10]. Nonetheless, there is declining trust towards healthcare and a patient perception of deteriorating accessibility [11, 12]. In addition, patients in Sweden experience more barriers in access to primary healthcare centres (PHCs) than other countries [13]. One factor partly responsible for this is the existence of two separate legal acts. One general healthcare act states that those with the greatest need are to be treated first, but a patient act states that healthcare should be accessible, including a guarantee of care. This constitutes a probable aggravating factor in regards to different interpretations by a healthcare worker and patient.

From a healthcare perspective, young adults (age 18–29 years) have been identified as an explanatory factor for many avoidable visits [4, 14]. On the other hand, young adult patients generally perceive more obstructing factors to self-care than to seeking healthcare [5]. A longitudinal study spanning 25 years showed that self-rated health has become poorer among the youngest age groups [15]. Social and economic forces, lack of work opportunities, and increased costs for independent living have prolonged entry to adulthood. Young adults are in a critical stage of their life course, often referred to as emerging adulthood [16]. No other life stage, except infancy, presents more complex changes on a personal, social, emotional, and developmental level. How young adults achieve stable adulthood is dependent on personal, family, and social resources they possess when entering this stage [16]. Going into a new stage in life, many young adults have to confront their first healthcare experience without an adult advocate. Therefore, this age group and stage in life are relevant to consider in healthcare research. Research concerning transitioning from adolescent healthcare to adult healthcare having chronic diseases is well known; knowledge, confidence, and independence have been highlighted as facilitating characteristics, but little is known about young adults’ healthcare utilisation and transition from adolescent healthcare in general [17–19]. Therefore, the aim of the present study was to explore how young adults perceive seeking first-line healthcare.

## Methods

### Design

The present study uses a qualitative grounded theory design according to Glaser as the method of analysis in an attempt to develop an explanatory theory on seeking first-line healthcare [20, 21]. The regional ethic review board in Linköping (2015/349-31) approved the study. All participants gave informed consent either recorded verbally or written.

### Setting

**Fig 1:**
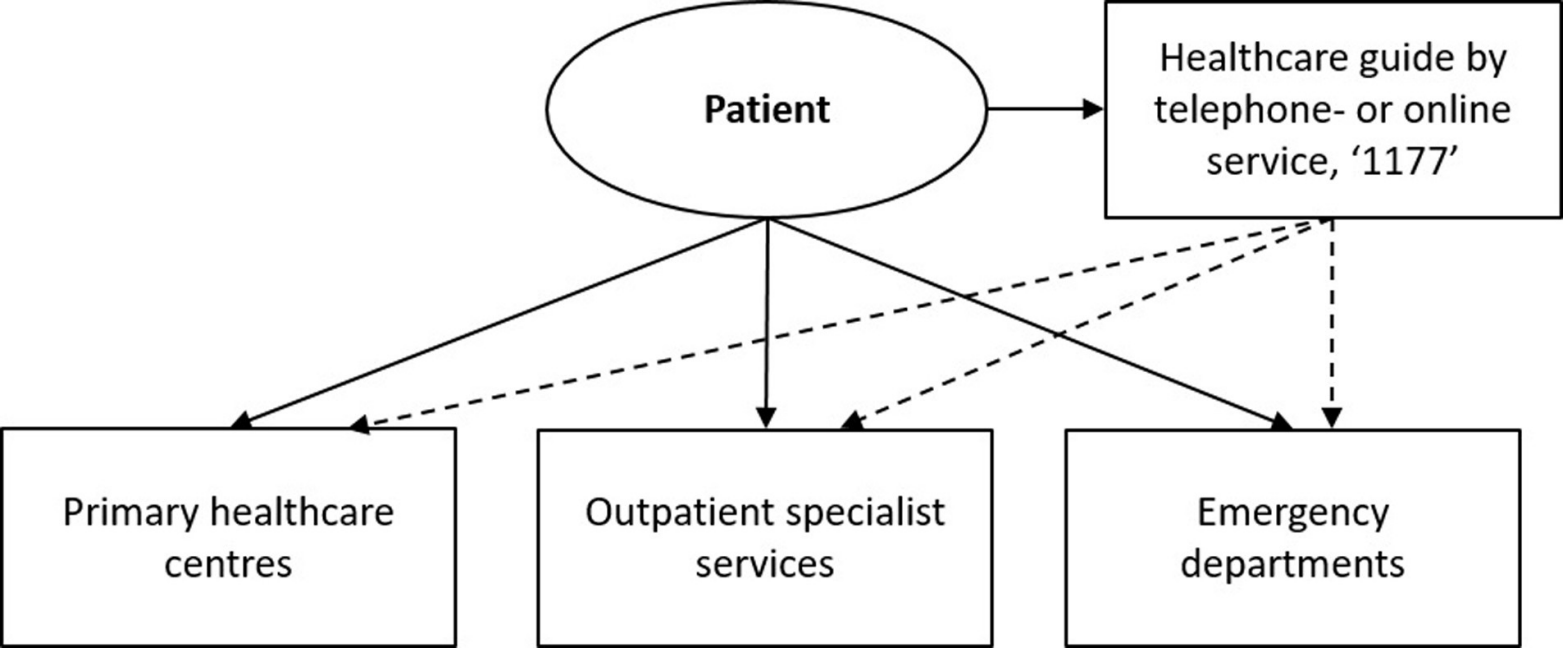
First-line care in the Swedish healthcare system. Structured for the majority to seek care at primary healthcare centres, preferably after contacting 1177. The dashed lines symbolize how 1177 in turn can refer the patient if considered necessary.

Swedish healthcare is publicly funded and, by law, committed to providing equitable healthcare to all citizens based on medical need [22]. Since 2014, the *Patient Act* has been in place to enhance patient integrity, self-determination, and participation [23]. Swedish healthcare is decentralized into 21 regions, and this study took place in three regions in Southeast Sweden. This study examines first-line care, which is defined as the first possible entrance to Swedish healthcare (Fig 1).

### Study population and data collection

Data were collected using theoretical sampling; data collection and analysis were carried out in parallel in six steps, with analysis after each step, all in accordance with grounded theory (Table 1). This enabled modification and provided the researcher with insight on changes needed in data collection to answer the objective.

**Table 1:** The process of theoretical sampling conducted in this study.

		Step 1	Step 2	Step 3	Step 4	Step 5	Step 6	Total
**Interviews**		**3**	**-**	**-**	**-**	**3**	**2**	**8**
**Observations**		**-**	**2**	**3**	**1**	**-**	**-**	**6**
**Distribution:**								
*Gender*								
	Male	1	1	1	1	1	-	**5**
	Female	2	1	2	-	2	2	**9**
*Age*								
	20–24 years	3	1	2	1	3	1	**11**
	25–29 years	-	1	1	-	-	1	**3**
*Department*								
	PHC	2	2	3	1	-	-	**8**
	ED	1	-	-	-	3	2	**6**
*Region*								
	Östergötland	3	2	-	1	1	-	**7**
	Kalmar	-	-	3	-	1	2	**6**
	Jönköping	-	-	-	-	1	-	**1**
*Main complaint*								
	Abdominal pain	1	1			2		**4**
	Injury/trauma	1		1		1	1	**4**
	Respiratory	1	1		1		1	**4**
	Skin			1				**1**
	Psychological			1				**1**
*Tried self-care*								
	Yes	1	2	2	1	2	1	**9**
	No	0		1				**1**
	n/a	2				1	1	**4**
Experienced with healthcare*								
	Yes	1	1	2		2	1	**7**
	No	2	1	1	1	1	1	**7**

Analysis was made after each step of data collection.

*Experienced with healthcare refers to having had multiple previous healthcare visits as an adolescent and/or adult.

The data consist partly of individual interviews, four conducted face-to-face and four conducted by telephone, and partly of observations with subsequent interviews. The period for data collection was April 2017 to June 2018. As participants were recruited from an earlier study [2], the inclusion criteria followed the requirements of that study: age 20–29 years, speaking and understanding the Swedish language, and seeking care for a minor injury or disease at that particular healthcare visit. Thereafter, theoretical sampling was performed.

The research team developed an interview guide for the interviews, which comprised open-ended questions followed by more direct questions when appropriate: “*I would like you to think back to your latest doctor visit and please share your experience in as much detail as possible*.” “*What where your thoughts before the visit*?*”* “*What where your thoughts after the visit*?*”* “*How would you describe your expectations when you called your healthcare centre*?*”* “*What do you consider to be good healthcare*?*”* For the interviews that followed observations, the observed healthcare visit guided what questions were asked in combination with the questions from the interview guide. For the observations, the researcher sought to be open-minded, letting the physician-patient relationship guide what they observed. For that reason, no observation template was used.

A total of 14 informants participated in the study (Table 1). After three opening face-to-face interviews, observations were added to enrich the data and enhance data collection. To gain a deeper understanding of the observed material, a later interview was conducted with each of the observed patients for follow-up questions concerning the observation. Depending on the data collection method, informants were chosen in various ways. For most of the interviews, participants had been asked in the previous study [2] about participation and those who agreed were contacted either by telephone or e-mail, after which a time and place were decided on for the interview. The face-to-face interviews took place in an undisturbed room at the research unit in accordance with the wishes of the informants. For the observations, patients visiting a PHC who matched inclusion criteria on one of the six days an observer (L.V.) was on site were asked to participate. Participants for the last two interviews were found by asking patients who matched the inclusion criteria and visited an ED whether they wanted to participate in an interview. One of the authors (I.W.) then contacted the patient, and a time and place for the interview was decided upon. The interviews lasted between 20 and 50 minutes. Interviews performed after an observation lasted 10 to 20 minutes. The observations lasted 30 minutes to 1 hour.

Interviews were performed by two of the authors (L.V., I.W.) and a research assistant; one is a public health scientist and two are registered nurses. All interviewers had previous experience with interviewing. All interviews were recorded and transcribed, and the observer took notes during all observations and dictated thoughts and ideas that were later transcribed. Data collection ended when theoretical saturation was achieved and no new insights on the material could be added.

### Analysis

Data were analysed by authors L.V., P.Y.U., E.T., and I.W. using grounded theory according to Glaser’s constant comparative analysis [20]. All transcribed interviews, together with notes and dictated observations, were printed in full and read several times. Initial coding was made by open substantive coding, identifying units of meaning in each interview and observation. In the next step, codes were evaluated and sorted into categories by selective coding. In the last step, categories were analysed and a core category finally emerged. Memos were written parallel to coding and served as support for further coding and data collection. After three initial interviews, the first open coding took place. During coding and later development of categories and the core category, a constant comparison was carried out between codes and categories, as well as within transcripts. In the process of theoretical coding, coding for how substantive codes interact, the categories and codes related to the core category were sorted, and a theory emerged.

## Results

**Fig 2:**
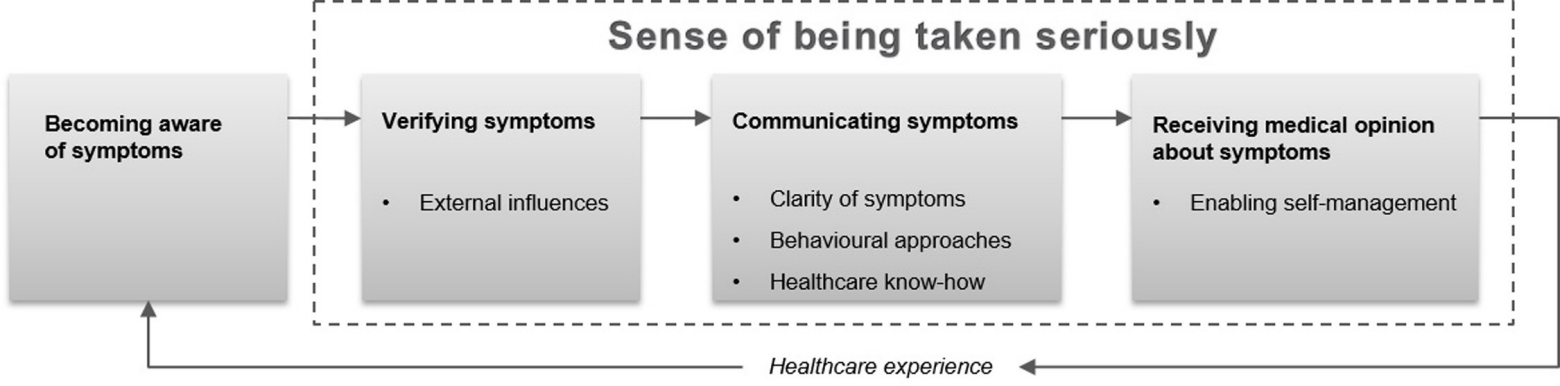
The process of seeking first-line healthcare among young adults. The process from becoming aware of symptoms to receiving a medical opinion about the symptoms. The dashed line highlights the part of the process that affects the patient’s sense of being taken seriously. Healthcare experience emerges after every healthcare visit, influencing every part of the process in future healthcare visits.

The categories were abstracted to a theoretical level as illustrated in Fig 2. The core category was the sense of being taken seriously.

### Sense of being taken seriously

Young adults seeking healthcare had an emergent concern: the sense of being taken seriously by their healthcare provider. The healthcare-seeking timeline contains four parts: *becoming aware of symptoms*, *verifying symptoms*, *communicating symptoms*, and *receiving a medical opinion about the symptoms*. In the process of seeking healthcare, communicating symptoms is often the most time-consuming part, but each part is equally important for young adults to sense being taken seriously.

The sense of being taken seriously develops during the healthcare visit and is dependent on more than just finding the cause of symptoms and being given treatment. The core category is comprised of experiences during and after the healthcare visit, including being treated with respect, being listened to, being taken care of, getting information, possible explanations, and a feeling of having gone in depth with probable cause while having the opportunity to speak freely and feel involved in the process. When young adults feel that they are not taken seriously in the healthcare setting, they describe feeling a need to justify their visit, almost as if they have to defend imagined symptoms.

#### Becoming aware of and verifying symptoms

Seeking healthcare is preceded by becoming aware of symptoms. Depending on how apparent the symptoms are, young adults verify symptoms by themselves or reach out to others, such as healthcare guides (1177), concerning care needs. Previous healthcare experiences influence whether patients verify symptoms on their own and the amount of time spent before seeking healthcare.

*External influences*. Unless the symptoms are apparent or severe, young adults reach out for advice from either health service information or family or friends. These persons play an important and sometimes conclusive role in whether young adults make a healthcare visit. Telephone- or web-based healthcare services are frequently used, and young adults embrace and think highly of the advice provided by these services. Family and friends or other social support often work as encouragement, but social support during a healthcare visit can be helpful in achieving the sense of being taken seriously. External influences also include reaching out to others with prior experience and/or with the same symptoms.

*Well*, *I remember that I rang the healthcare guide first and talked to them*, *because I had a stomach ache–it had been going on for several hours and I was starting to get a bit worried*. *And she thought that if it hadn’t gone in an hour or so then I should go to A&E*. (Interviewee #3)

#### Communicating symptoms

The ability to communicate symptoms is the most crucial part of seeking healthcare for young adults. Clarity of symptoms, behavioural approaches, and healthcare know-how are essential for managing and communicating symptoms.

*Clarity of symptoms*. Young adults seek healthcare for a variety of reasons and with more or less distinct symptoms. Clarity of symptoms is the chief category when communicating symptoms; the clearer the symptoms are, the less the patient needs to convince the doctor about their need for care. In addition, healthcare know-how and behavioural approaches become less important when symptoms are obvious.

*Behavioural approaches*. In the presented theory, young adults exhibit four different behavioural approaches: submissive, conformable, insistent, or assertive. In the case of a first-time visit, the patient is more submissive and has a lack of opinion, leaving most of the communication to the doctor. A conformable appearance is attributed to patients with limited healthcare experience. The little experience they may have is positive, making them likely to rely on the healthcare system and their doctor’s competence. The insistent patient has had previous negative experiences within the healthcare system (i.e., the sense of not been taken seriously by healthcare personnel in the past). Their confidence in healthcare has been damaged and their trust in doctors’ competence has been negatively affected. Young adults who are assertive (i.e., have confidence that is not a threat to others) have experienced previous positive healthcare visits and are already patients due to other, often chronic, diseases. They can have either clear or unclear symptoms but possess healthcare know-how. Assertive patients yield better-perceived healthcare visits, partly because of previous healthcare experience, already being in the healthcare system, and prior healthcare knowledge. Young adults with an assertive behavioural approach are most likely to be taken seriously and, therefore, are most satisfied with their healthcare visit.

*That they can’t walk all over me as easily anymore*. *It feels like I always have some good counter-argument–or at least I usually do*. *When I go there*, *I’ve read quite a lot about it* … (Interviewee #10)

*Healthcare know-how*. When communicating symptoms, healthcare know-how is one factor that increases when one’s symptoms are less clear, as it forces the patient to be well-read and to become an expert on his or her own symptoms while learning how the healthcare system works. Healthcare know-how is about learning what has not been explicitly stated. By learning what healthcare personnel look for and knowing the underlying question, the patient gets one step ahead and increases the likelihood that he or she will provide the needed information. This kind of tacit knowledge evolves among patients with previous negative experiences and particularly when symptoms are unclear.

*I sometimes feel that you have to be almost* … *well*, *you almost have to exaggerate*. *That they don’t really take you seriously*. (Interviewee #10)

#### Receiving a medical opinion about symptoms

The concluding part of the healthcare-seeking process is receiving a medical opinion about symptoms and helping the patient self-manage. Preventive secondary care is the category described below. Receiving a medical opinion about symptoms is a passive step for the patient, unlike actively communicating symptoms. In this part of the process, the doctor gives his or her conclusion with little input from the patient. In this last step, patients expect the doctor to explain how their symptoms may be self-managed. The time given for receiving a medical opinion about their symptoms affects a patient’s opinion of healthcare and, thereby, their healthcare experience. The more that healthcare personnel enable the patient to self-manage symptoms, the less negative the healthcare experience seems to be for the patient.

*The doctor explains what it is and that they don’t know the cause*. *He explains what the illness is like and how it starts*, *and while he’s talking*, *you can see yourself that it sounds likely that that’s what it is*. *The patient gets a leaflet about the illness and is told that it will pass*. (Observation #14)

*Enabling self-management*. The term self-management refers to secondary prevention and is used instead of “self-care” to make a distinction between preventive care (i.e., taking measures not to seek healthcare) and preventive secondary care (i.e., being able to handle the symptoms continuously on one’s own). The degree of commitment and effort appears to differ depending on the individual, but enabling self-management in this context is about facilitating the essential factors that apply to all patients in recovery, ranging from understanding how to manage with prescribed medication to information on what to do if symptoms return. There is an expressed need to know what you suffer from and get information about what actions to consider in the future. For example, the patient wants to know whether to try to get to the bottom of the symptoms, be suggested a follow-up, or given information that may prevent further problems instead of hearing, “We were unable to find anything.” In these cases, the feeling of being abandoned and deluding oneself is implied.

… *to be involved in your care plan*. *To* … *[sighs]* … *More time with a doctor so that you can understand why and how this happened and what to do to stop it happening*, *and what I can do to make it better*. (Interviewee #5)

*Healthcare experience*. In general, young adults lack the experience of attending a doctor visit on their own. Of those who do have prior healthcare experience, they either acquired it through other types of issues—predominantly chronic diseases—or previously sought help for the same symptoms.

Healthcare experiences permeate each step of the healthcare-seeking process and foster the ability to communicate symptoms (i.e., a patient’s behavioural approach and healthcare know-how develop). Previous negative experiences affect patients, causing them to wait longer to seek care, question healthcare personnel’s competence, and develop a behavioural approach of being insistent. On the other hand, with positive experiences, patients’ behavioural approaches become more conformable and can, by extension, become assertive.

…*and so when you meet new doctors*, *it’s a bit of a mental process*. *A bit like ‘OK*, *how much of a struggle will it be this time*?*’* (Interviewee #12)

## Discussion

In this study, a sense of being taken seriously was the main concern for young adults when seeking healthcare. This is likely an important concern for all age groups; however, with regard to this study’s results indicating every single healthcare visit influences future visits, this may play a more important role for young adults because their experiences significantly form their future feelings towards healthcare. Earlier research on young adults’ healthcare-seeking behaviour from the patient perspective is scarce, which is one reason for using grounded theory as a method. However, similar conclusions can be found in recent research indicating that young adults have a need to be heard, and that a lack of recognition contributes to feelings of marginalisation, with a negative impact on psychological factors [24]. The need to be taken seriously illustrates young patients’ desire to be seen as ‘a whole person’ and not solely as a symptom, but also that young adults perceive difficulties in being treated as equal adults and feel a need to justify the cause of their individual visits. Being taken seriously permeates and affects all stages of the healthcare visit, thereby influencing young adults’ overall satisfaction and the result of the healthcare visit process. When being treated as equal, with attention to personal circumstances, being met with empathy, and exploring symptoms in depth, patients feel they are being taken seriously to a greater extent [25]. By extension, the results imply a need for healthcare to centre on the person, not solely the symptoms, and apply a person-centred approach from the first outpatient healthcare visit. Person-centeredness is a question of recognising a patient’s needs, who they are together with what they need, building a partnership, seeing the patient’s uniqueness, and empowering the patient with respect to autonomy [26]. By extension, for healthcare providers, this would mean working with the more known multi-dimensional concept of person-centred care [27]. By establishing more person-centred care in meeting with young adults in first-line care, conditions can be created that benefit both healthcare personnel and patients: an extended sense of being taken seriously and better conditions for the next healthcare visit. For example, instead of just expressing that they are ‘unable to find anything’, creating the feeling of being abandoned, five extra minutes can be spent discussing a plan with the patient, such as the patient’s thoughts and feelings about these non-results, with a focus on managing future self-care, which could increase confidence in healthcare.

As young adults enter emerging adulthood [16], they try to go from being dependent children to independent individual adults, and are influenced by their social, cultural, and physical environment. Different exposures in these environments during the first life stage as an adult could significantly influence later outcomes in life [16]. Young adults are an age group that has been shown to perceive the most age discrimination of all age groups, and old age is shown to be a protective factor against perceiving barriers to PHC [28, 29]. With negative healthcare experiences, young adults may foster an unwanted behavioural approach and wait longer to seek care. As shown in previous research, patients experiencing low levels of patient-centred communication with their healthcare provider are less likely to seek needed care, and as many as one-third of adults avoid healthcare visits despite their own belief that care is needed. Experiential and emotional traits, together with interpersonal communication, are considered to be important. In addition, connectedness has been shown to be more important for patients, rather than more formal participation in the decision [27, 30, 31].

The sense of being taken seriously could also be linked to health literacy—that is, a patient’s capacity to access, understand, appraise, and apply health information, as health literacy is linked to access to care [32]. Sufficient health literacy helps patients exert control over care (i.e., organizing care, interacting with providers, and performing self-care) [33]. Health literacy is also a factor when seeking healthcare in regards to approachability (i.e., comprehending information, identifying services, and participating in outreach activities) [34]. Earlier research has shown that patients with insufficient health literacy have lower reliance on healthcare systems [2]. If healthcare wants to change how young adults seek care, it is important to foster trust, reliance, and transparency [35]. The present results suggest that patients with a behavioural approach of being assertive also possess much healthcare know-how and probably have sufficient health literacy. This indicates the need for healthcare personnel to pay certain attention to patients with submissive, conformable, or insistent behavioural approaches when providing a medical opinion about symptoms.

Findings in this study indicate that young adults try to seek healthcare thoughtfully. When verifying symptoms, many of them choose to contact 1177 before visiting a PHC or ED. External influences, such as 1177 or family or friends, together with past experience, have been shown to affect the probability of young adults seeking healthcare. This is also supported in other studies; the need for encouragement and support and healthcare personnel’s advice is highly prioritized [19, 36]. Today’s healthcare service in Sweden emphasises self-care based on the considered need to decrease healthcare utilisation, but young adults have difficulties performing self-care instead of seeking healthcare [5]. It could be argued that young adults present an imbalance between self-care demands and self-care prerequisites, seen here as external self-care demands contra internal self-care prerequisites [37]. Self-care is defined as an individual’s self-initiative performed on their own behalf, and is often referred to as taking one’s own measures when symptoms are simple, thereby avoiding seeking healthcare [37]. The degree of self-care managed by an individual is dependent on knowledge [5]. The study’s theory shows willingness to handle continued care on one’s own, as enabling self-management is requested by patients. Giving young adults increased knowledge about their condition during the healthcare visit could potentially increase future self-care, reducing revisits. In addition, when giving a patient the right prerequisites, healthcare experiences become more positive, which could contribute to better conditions for future healthcare visits.

The theory that emerged from the data in this study does not capture all of the different components possibly influencing when young adults seek first-line healthcare, but emphasizes major components of relevance in the studied setting that could be transferable to similar settings. The interviewers and analysts are all healthcare employees in Region Östergötland and Kalmar. They are registered nurses and a public health scientist, none of whom have a relationship with any of the studied patients. They all possess previous experience with qualitative data collection and analysis.

A strength of this study is the adherence to criteria concerning grounded theory [20]. Participants were diverse and, by using both interviews and observations, relevant results were enabled. Data were analysed in parallel with data collection, facilitating possible adjustments as the data collection continued. This amplifies the probability of the theory fitting the data. The developed theory explains what is happening, can predict what is about to happen, and interprets special events within a certain part of the process, all of which are important for a theory to be workable. The presented theory is concise, but not solid, and therefore modifiable. Another strength is the triangulation [38] in which three researchers were included in the process of analysis with several revisions before the final theory emerged.

A limitation of the study is that observations were made in PHC settings. Conducting observations in healthcare settings is often time-consuming and sensitive, concerning ill and potentially fragile persons. Considering the sensitivity of the topic, in addition to the population of interest often having long wait times at EDs, the decision was to limit observations to PHCs. The research team made the assessment that it had a minor influence, as several of the interviews still captured ED experiences and the main aim of the observations was to capture young adults’ behaviour in meetings with a doctor in first-line care; the meeting place was considered less important.

## Conclusion

This study reflects several insights in the healthcare-seeking process from a young adult perspective. The sense of being taken seriously appears to be young adults’ main concern, developing both during and after the visit and being dependent on more than just finding the cause of symptoms and being treated. Healthcare providers should place greater focus on the final step in the healthcare-seeking process–delivering a medical opinion about symptoms. Investing a few extra minutes to give support for future self-care regardless of diagnosis may increase positive healthcare experiences and increase future self-care among young adults.

## Supporting information

S1 File Interview guide. (PDF)
