# Supplementary material for: Young adults’ needs when seeking first-line healthcare: A grounded theory design
Source: PLoS One. 2022 Feb 15;17(2):e0263963. doi: 10.1371/journal.pone.0263963 (PMC8846516; doi:10.1371/journal.pone.0263963)
Supplement: S1 File — (PDF) [file pone.0263963.s001.pdf]

S1 Interview guide

Background questions:

- Name
- Residence
- Occupation (working, studying, etc.)

Interview questions:

I would like you to think back to the last time you visited a doctor at the health centre or the emergency department.

1. **Tell me about the visit in as much detail as you can.**
   Post-observation interview: What did you think about the visit?
   (the reason for your visit, what type of healthcare facility you visited, how you felt about the contact, what you thought of the doctor)
   - How do you feel the appointment went?
   - What is your overall impression of the appointment?
   - Can you think back and perhaps give some examples of when a visit didn't seem as good/seemed better? How would you describe the difference between these two visits?

2. **What were your thoughts before the visit?** (feelings, concerns)
   - What information did you have before your visit? search for information
   - the steps to seeking care

3. **What were your thoughts after the visit?** (feelings, concerns)
   - information from the doctor
   - satisfied, helped

4. **Could you describe your expectations when you call a health centre?**
   How would you describe yourself as a patient?

5. **When do you think someone should visit a health centre or an emergency department? Do you think it is clearly communicated to the general public when someone should seek treatment and which facility they should visit?**

6. **How would you describe the health centre you are registered with?**
   Did you actively choose it or were you assigned to it?

7. **What constitutes good healthcare for you?**
   What is most important for you?
